# Supplementary material for: Predicting Intention to Receive COVID-19 Vaccination in People Living with HIV using an Integrated Behavior Model
Source: Vaccines (Basel). 2023 Jan 29;11(2):296. doi: 10.3390/vaccines11020296 (PMC9962027; doi:10.3390/vaccines11020296)
Supplement: Supplementary file 1 [file vaccines-11-00296-s001.zip › vaccines-2115239-supplementary.pdf]

Table S1. Analysis on items of each IBM component.

Items	r	B (p value)	R	R^2
**Instrumental attitude**			0.497	0.232
As long as I take ART regularly, I will be as healthy as non-HIV individual	0.607	0.068 (0.209)		
To end the pandemic, I will still follow the health protocol after COVID-19 vaccination	0.821	-0.021 (0.789)		
Getting COVID-19 vaccination means I can protect my family	0.780	-0.036 (0.672)		
Getting COVID-19 vaccination means I can protect myself	0.761	0.070 (0.369)		
Vaccination against COVID-19 is one of the efforts to end the pandemic	0.835	0.162 (0.062)		
Getting COVID-19 vaccination means I contribute to create herd immunity	0.832	0.102 (0.233)		
COVID-19 vaccination can develop my immunity against COVID-19 infection	0.527	0.068 (0.206)		
Getting COVID-19 vaccination will reduce the severity of SARS-CoV-2 infection once I get infected	0.645	0.199 (0.001)*		
**Subjective norm**			0.644	0.405
I will receive COVID-19 vaccination because it is the government program	0.648	0.150 (0.002)*		
My work colleagues expect me to receive COVID-19 vaccination	0.824	-0.027 (0.685)		
My doctors expect me to receive COVID-19 vaccination	0.810	0.166 (0.011)*		
My senior expects me to receive COVID-19 vaccination	0.814	0.128 (0.060)		
The religious leader that I respect expects me to receive COVID-19 vaccination	0.766	0.150 (0.012)*		
My family expect me to receive COVID-19 vaccination	0.743	0.202 (0.001)*		
**Perceived of behavioral control**			0.427	0.178
How often you feel in ease situation to achieve your wish?	0.555	0.110 (0.036)*		
How often your confidence "I can do it", dominate your daily activity?	0.555	0.360 (<0.01)*		

*Significance
